# Evaluation of patients on sertindole treatment after failure of other antipsychotics: A retrospective analysis

**DOI:** 10.1186/1471-244X-8-16

**Published:** 2008-03-14

**Authors:** Jean-Michel Azorin, Susana Murteira, Karina Hansen, Mondher Toumi

**Affiliations:** 1 Department of Psychiatry, CHU Sainte Marguerite, Marseilles, France; 2 Global Outcomes, Risks and Market Access, H. Lundbeck A/S, Paris, France

## Abstract

**Background:**

Use of the atypical antipsychotic sertindole was suspended for four years due to safety concerns. During the suspension, the regulatory authorities required further studies, including this one, to be conducted. The purpose of this study was to determine if a subset of patients with psychotic illness exists which particularly benefits from sertindole treatment after failure of other antipsychotic drugs, including atypical antipsychotics.

**Methods:**

This was a retrospective single-arm observational crossover study of 344 patients, who served as their own controls. Patients mainly from the Sertindole Safety Study who had shown good response to sertindole, and who had followed up to four alternating six month periods of treatment with sertindole and other antipsychotics, were included. (In Period 1 patients took non-sertindole treatment, in Period 2, sertindole was taken, in Period 3, patients reverted to non-sertindole treatment, and in Period 4, sertindole was taken again.) Patient records for each period of treatment were assessed for objective data: number and duration of hospitalizations due to worsening of psychotic symptoms; the amount of self-harming behaviour; indicators of social status. Retrospective evaluation of changes in clinical symptoms from the patients' records was also conducted. Dates and reasons for stopping and/or switching medication were also recorded.

**Results:**

There was improvement in all objective measured parameters during the periods of sertindole treatment. In particular, the average number of hospitalizations per year due to worsening of psychotic symptoms was reduced in the following way in the group studied over four treatment periods: Period 1 (non-sertindole treatment) 3.4; Period 2 (sertindole treatment) 1.0; Period 3 (non-sertindole treatment) 2.0; Period 4 (sertindole treatment) 1.8. The duration of hospitalizations also decreased significantly during the periods of sertindole treatment. Results showed that patients improved in objective social parameters when switched to sertindole treatment; assessment of the patients' affective lives showed a significant increase in the number of patients having a stable relationship during sertindole treatment; and assessment of the number of patients employed showed an increase after the first and second switch to sertindole treatment (from Period 1 to Period 2 and from Period 3 to Period 4, respectively).

Adverse events and lack of efficacy were the main reasons for switching to sertindole.

**Conclusion:**

A group of patients benefited from sertindole after other antipsychotic treatments, including that with atypical antipsychotics, had failed. Further studies are needed to investigate if there is a specific patient profile that corresponds to these responders.

## Background

Schizophrenia is a chronic, complex, and heterogeneous disease that affects most aspects of psychological functioning. The disease is frequently associated with cognitive and depressive symptoms and commonly manifests at an early adult age. It can have a devastating impact on familial, social and vocational aspects of patients' lives [1-3]. Long-term treatment with antipsychotic drugs is the major factor in preventing relapse [4-6].

For the patient suffering from schizophrenia it is crucially important to receive the best possible treatment. The individual responses to different treatments are highly variable, and therefore a wide selection of clinically efficacious and safe drugs will maximize the chances that the appropriate treatment is eventually prescribed to each patient [7,8].

Although clozapine has been a great improvement in addressing unmet medical needs for treatment-resistant patients, it is still thought that many patients with schizophrenia do not respond to any available antipsychotic treatment [9,10].

Sertindole is an atypical antipsychotic drug indicated for the treatment of schizophrenia. It was first authorised in the United Kingdom in 1996 and subsequently in other European countries. Due to concerns related to cardiac safety which arose after its launch, the marketing of sertindole was suspended by Lundbeck in November 1998 [11].

In June 1999, the European Committee for Proprietary Medicinal Products (CPMP, now the CHMP) adopted an Opinion recommending the temporary suspension of the Marketing Authorizations for sertindole and asked for a complementary benefit/risk evaluation of the product [12]. The present study (the "NICHE" study) was part of this evaluation. It was performed in order to respond to the question of whether there is a subset of patients that clearly improve during sertindole treatment after failure with other antipsychotics.

This observational study was designed in order to include patients who had two non-consecutive treatment periods with sertindole. Since market availability of sertindole was restricted after the suspension, patients were mainly selected from those who were identified in a previous survey, the Sertindole Safety Survey [13] therefore giving the name the present study, the NICHE study.

The NICHE study provided an opportunity to document the improvement, or otherwise, of a subset of patients who had benefited from sertindole treatment, and for whom alternative therapies had not shown comparable results. The assessment of safety was not an objective of this study, as this was addressed in the Sertindole Safety study.

## Methods

### Data sources

The 344 patients included were mainly from the European countries that had launched sertindole before its marketing suspension in 1998. The only exceptions were patients from France and the UK (where sertindole had not been launched). For these latter countries, patients that had been included in long-term clinical trials and who fitted the predefined profile were selected, accounting for 5.6% of the patients in the present study (Table 1).

**Table 1:** Total number of patients per country

**Country**	**Number of patients n (%)**
Austria	18 (5.2%)
Belgium	54 (15.7%)
Czech Republic	12 (3.5%)
Estonia	2 (0.6%)
Finland	7 (2.0%)
France	15 (4.4%)
Germany	97 (28.2%)
Hungary	55 (16.0%)
Latvia	71 (20.6%)
Netherlands	9 (2.6%)
United Kingdom	4 (1.2%)
Total	344 (100%)

In order to include patients who had two non-consecutive treatment periods with sertindole, patients were selected from those who had been treated through the named-patient program (set up after sertindole suspension), and who were identified in a previous survey, the Sertindole Safety Survey.

The Sertindole Niche Survey is a retrospective study, with no patient exclusion criteria and no personal identification required. For this study there was no need for ethics committee approval of the study according to the countries regulations at the time of conduction of this study in Austria, Belgium, Czech Republic, Estonia, Finland, France, Germany, Hungary, Latvia, Netherlands and United Kingdom.

The Sertindole Niche Survey was based on the Sertindole Safety Survey, for which local regulatory rules were followed in each participating country. Where applicable, the Sertindole Safety Survey study was submitted to and approved by the relevant authorities and/or local ethics committee/institutional review board.

Patients were 18 years or older; their primary diagnosis was that of a psychosis, mainly schizophrenia.

**Figure 1:**
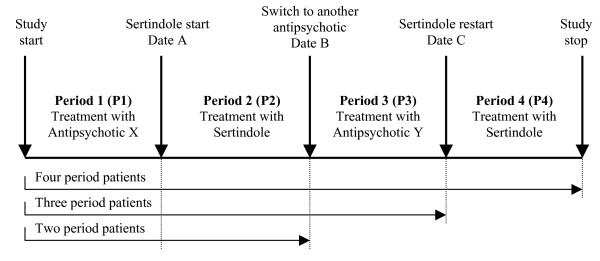
Types of patients and study design.

A treatment period of at least six months with the concerned antipsychotic was required (see Figure 1). In the pre-study initial selection, patients were excluded if they experienced relapse under sertindole treatment. The patient population encompassed four possible periods of antipsychotic treatment (periods 1 to 4, of which two were with sertindole and two were with other antipsychotic drugs), and three groups of patients (patients who went through 4, 3 or 2 periods of treatment, respectively).

The age, gender, DSM IV classification, history of psychiatric disorder, general medical history, and history of drug abuse of the patients were recorded before the evaluation.

All antipsychotic medication taken within the observational period was recorded, including the type of drug, the indication, the dose, the start and end date of treatment, and the reason for stopping or switching medication. Concomitant medication was not recorded.

### Study design

This was a single-arm, retrospective, crossover study. Data collection was not blinded and treatments had not been randomly allocated. The patient served as his or her own control.

All patients followed alternate periods of antipsychotic treatments: after an initial treatment period on a non-sertindole antipsychotic regimen of at least six months, patients who either were not responding or were experiencing tolerability problems, were switched to sertindole treatment (Figure 1). Following sertindole's market suspension, patients were switched again to other antipsychotics. Subsequently, some of the patients went on to receive a further period of sertindole treatment, under the named patient compassionate use programme. Up to four treatment periods of around six months each were thus studied. This design thus produced three separate patient groups from the initial 344 patients: a group of patients that had experienced only two treatment periods, a group of patients that had experienced three treatment periods, and a group of patients that had experienced four treatment periods.

### Data analysis

For each treatment period, patient records were assessed for objective parameters (the number of hospitalizations and the amount of self-aggression behaviour [self-harm and suicide attempts]). These data are supportive of the retrospective data on patient clinical symptoms (measured by the Clinical Global Impression and Global Assessment Function scales).

The psychiatrist attending each patient retrospectively assessed the longitudinal course of the psychosis using the five main symptoms defined in the DSM IV for psychosis longer than one year (all types considered). These symptoms were rated from 0 to 3 according to intensity (0 = absent, 3 = severe). In addition, the following assessments and records were used to support the data:

• Clinical symptom evaluation was based on the Clinical Global Impression (CGI) and the Global Assessment Function (GAF) scales.

• The number of hospitalizations and the number of days in hospital were recorded.

• Self-harming behaviour was assessed by the number of suicide and self-harm attempts made.

• The presence of a stable personal relationship and the employment status provided objective information in support of the social assessments.

### Statistical analysis

The statistics were essentially descriptive. The results were analysed taking into account the score of each individual item, enabling the comparison of the results between different periods.

Number of hospitalizations, duration of hospitalization, and number of self-harm and suicide attempts were computed by treatment period. Data were analysed using SAS software (version 6.12). Quantitative variables were expressed as mean ± standard deviation (SD), while qualitative variables were presented using frequencies and percentages. Chi-square tests (or Fisher's exact tests when appropriate), as well as t-tests (or Mann-Whitney tests when appropriate) were used to compare qualitative or quantitative data. All statistical tests were two-sided, with the α risk set to 0.05.

## Results

### Patients' profiles at start of study

344 patients were included in this study; 185 (53.8%) were women and 157 (45.6%) were men (gender was missing for 2 patients [0.6%]). Of the 344 patients, 57 had followed four treatment periods (P1–P4). The average age (± SD) was 39.3 (± 14.2) years. The average duration (± SD) of the present psychiatric disorder was 11.8 (± 9.1) years. See Table 2 for the patients' baseline characteristics.

**Table 2:** Patient clinical profile at baseline

**Patients' baseline profile**	**Number of patients n (%)**
**Primary diagnosis**	
Schizophrenia*	**304 (88.6%)**
Paranoid type	218 (71.9%)
Disorganized type	31 (10.2%)
Undifferentiated type	29 (9.6%)
Residual type	15 (5.0%)
Catatonic type	10 (3.3%)
Other than schizophrenia	**39 (11.4%)**
Schizo-affective disorder	19 (51.4%)
**Concomitant mental disorders**	**105 (30.7%)**
Mood disorders	76 (72%)
Anxiety and other disorders	88 (83.8%)
**Hospitalization due to psychosis**	**326 (94.8%)**
Last hospitalization duration > 1 month	204 (62.5%)
**Patients that had been physically violent towards another person at least once**	89 (25.9%)
**Patients that had attempted self-harm at least once**	68 (20.4%)

*data for 1 patient was missing

### Patients' profiles during the treatment periods

**Figure 2:**
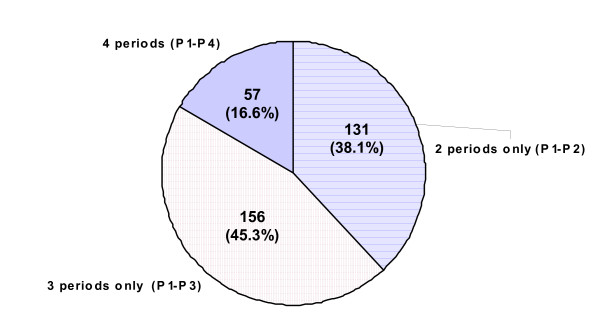
Patients per number of periods experienced.

From the patients included in this study, around 17% followed four treatment periods. From the remaining patients, around 45% followed three treatment periods and around 38% followed two treatment periods. The number of patients within each group is shown on Figure 2. After the first treatment with sertindole, the average treatment duration did not change significantly between periods, ranging from 8.7 ± 5.9 (SD) months in Period 2 to 6.4 ± 4.3 (SD) months in Period 4, as shown in Table 3.

**Table 3:** Comparison of duration of treatment periods

	Period 2	Period 3	Period 4
Mean duration ± SD (months)	8.7 ± 5.9	7.1 ± 5.2	6.4 ± 4.3
*p*-value	0.244		0.465

Analysis of the types of antipsychotic drugs taken during Periods 1 and 3 (the non-sertindole periods) showed an increase in the percentage of atypical antipsychotics used over time (Table 4). The most prescribed antipsychotics in Period 1 were clozapine (24.6%) and risperidone (24.6%). In Period 3, the most prescribed antipsychotic was olanzapine (42.1%).

**Table 4:** Antipsychotic treatment during Period 1 and Period 3

	**Type of Antipsychotic**	**Period 1 n (%)**	**Period 3 n (%)**
**Four treatment period group (P1–P4)**	Only typical antipsychotics	29 (50.9%)	6 (51%)
	At least one atypical antipsychotic	28 (49.1%)	51 (89.5%)
**Three treatment period group (P1–P3)**	Only typical antipsychotics	64 (41.0%)	17 (11.0%)
	At least one atypical antipsychotic	92 (59.0%)	138 (89.0%)
**Two treatment period group (P1–P2)**	Only typical antipsychotics	14 (10.7%)	NA
	At least one atypical antipsychotic	117 (89.3%)	NA

The main reasons for switching to sertindole at the end of the first treatment period (P1) were found to be firstly, lack of efficacy, and secondly, experience of adverse events under the previous treatment.

The main reason for stopping sertindole after Period 2 was due to its market suspension or consequent difficulties in supply.

### Results from the group that followed four treatment periods (P1–P4)

**Figure 3:**
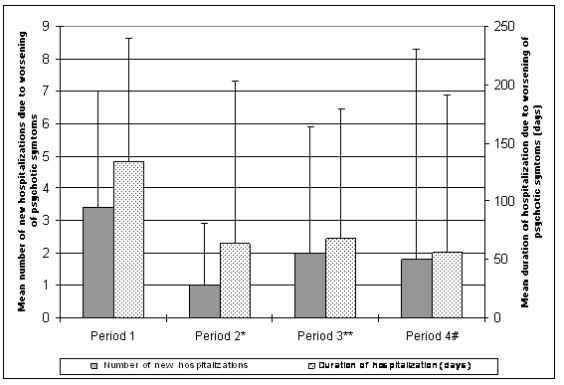
**Mean number and mean duration of hospitalizations due to worsening of psychotic symptoms in the four treatment period group**. Mean number of hospitalizations (comparison with previous period): * p < 0.001, **p = 0.02, ^#^p = 0.001. Mean duration of hospitalizations(comparison with previous period): * p < 0.001, **p = 0.14, ^#^p = 0.02.

The comparison between adjacent periods shows that the average number of hospitalizations due to worsening of psychotic symptoms was significantly decreased during the first and second period with sertindole treatment (P2 and P4, respectively). These findings are summarised in Figure 3. From Periods 1 to 2, duration of hospitalisation (± SD) was significantly reduced from 134 days (± 106) to 65 days (± 139) (p < 0.05). The duration of hospitalization increased to 68 days (± 108) in Period 3, although this was not statistically different from Period 2. From Period 3 to Period 4, a significant reduction in duration of hospitalization was also seen (68 days [± 108] in P3 to 56 days [± 135] in P4, p = 0.019, respectively).

The number of self-harm and suicide attempts was significantly reduced during both sertindole periods compared to the non-sertindole periods. After starting the first sertindole treatment the percentage of self-harm attempts decreased from 22.8% to 3.5% (p < 0.001), and during the second period of sertindole treatment it reduced from 5.8% to 1.8% (p < 0.05). The number of suicide attempts also followed this trend with a reduction from 15.8% to 3.5%, and from 7.0% to 0%, during the first and second sertindole-treatment periods, respectively.

Analyses of patients' affective lives and employment and occupational statuses showed that patients improved in objective social parameters when switched to sertindole treatment, i.e., in Periods 2 and 4 *(*see Figure 1). Assessment of the patients' affective lives showed a significant increase in the number of patients engaged in a stable relationship during sertindole treatment (54.4% in Period 2 and 57.1% in Period 4), compared with the periods of non-sertindole treatment (38.6% in Period 1 and 45.6% in Period 3) (Table 5). The number of patients employed more than doubled from Period 1 to Period 2 (from 14.0% to 31.6%) and also increased, although not statistically significantly, from Period 3 to Period 4 (from 22.8% to 33.9%) (Table 5).

**Table 5:** Objective Social Assessments

	**Period 1**	**Period 2**	**Period 3**	**Period 4**
**Patients' Affective Lives **Engaged in a stable relationship	22 (38.6%)	31 (54.4%)	26 (45.6%)	32 (57.1%)
*p*-value	0.003	0.059		0.014
**Employment and Occupational Status **Employed most of the time	8 (14.0%)	18 (31.6%)	13 (22.8%)	19 (33.9%)
*p*-value	0.008	0.096		0.014

Clinical evaluation data were also supportive of the above objective results: during sertindole treatment, i.e., in Periods 2 and 4 *(*see Figure 1), 26.3% and 21.4% of the patients, respectively, were found to be in full remission according to the DSM-IV criteria (Table 6). Clinical evaluation based on clinical scores showed that all the symptoms of current psychiatric disorders were on average at a higher level during Period 1 and 3 than during the sertindole-treatment periods (P2 and P4); during both Periods 1 and 3, the patients were on average moderately to markedly ill on the CGI scale, whereas during both Periods 2 and 4 they were borderline mentally ill to mildly ill. The mean GAF scores were 35.6 and 44.8 during Periods 1 and 3 respectively, and 61.4 and 65.5 during Periods 2 and 4, respectively.

**Table 6:** Remission profile in the four treatment period group

**Type of course**	**Period 1 n (%)**	**Period 2 n (%)**	**Period 3 n (%)**	**Period 4 n (%)**
Single episode in full remission	0 (0.0%)	5 (8.8%)	0 (0.0%)	8 (14.3%)
Full remission	0 (0.0%)	15 (26.3%)	2 (3.5%)	12 (21.4%)

## Discussion

The results show that there is a subset of patients who were switched to sertindole that had an improvement in their clinical symptoms, which was reflected in a decrease in the number and duration of hospitalizations, as well as suicide attempts.

When sertindole was first suspended from the market, it could only be prescribed under the named-patient program. Although some clinicians continued to prescribe sertindole, many patients were switched to other treatments. From clinical observation, it was suggested that there might be a subset of patients who respond uniquely to sertindole [13]. In order to identify whether this proposed subset of patients exists, we conducted this retrospective study of the health records of patients who had received alternate periods of non-sertindole antipsychotic drug therapy and sertindole therapy. This study was conducted using retrospective methodology, as sertindole was suspended at the time and it was not possible to run prospective clinical trials.

This study was not designed to compare antipsychotics, but to document, in a standardised way, if a subset of patients exists for whom multiple previous antipsychotic treatments had not been satisfactory, but who had improved with sertindole. As a result, the total number of patients to be included was not calculated. Because the study was conducted retrospectively, the treatment pattern and patient management were unaffected by the conduct of the study. However, the treatments were not randomly allocated and the reliability of the unblinded efficacy data may be questioned. Data collection was therefore standardised for the various assessment periods, using both objective criteria (number and duration of hospitalizations, number self-harm and suicide attempts, patient's affective life and employment and occupational status) and subjective criteria (the clinical scorings, GAF, CGI).

Each patient served as his or her own control, and comparisons were achieved in crossover periods in the patients who followed four treatment periods. The results from the group of patients who followed the four treatment periods provide the strongest possible indicator for the causal relationship between sertindole treatment and improvement in symptoms. This design could be compared to the challenge-dechallenge-rechallenge strategy in pharmacovigilance.

Results from the patients who followed only two or three alternate treatment periods, although confirming what was observed in the P1–P4 group, have not been reported here in detail, as they were not the focus of this article. It would be interesting to analyse the results between these groups in further studies. The individual clinical results have not been analysed statistically as the patients were not randomly selected (only patients who responded to sertindole treatment were included), and analysis would have had no value.

The improvements in all criteria during the sertindole treatment periods provide evidence of a sub-group of patients who respond particularly well to this drug. In particular, the reduction in number of hospitalizations due to worsening of psychotic symptoms provides objective evidence for this improvement, as hospitalization for worsening of psychosis has been demonstrated to be a good indicator of relapse in patients with schizophrenia [14].

Why a particular subgroup of patients responds to sertindole in this way is unknown, but it may be related to the specific chemical structure and the specific receptor profile which lead to the unique pharmacological profile of the drug [15]. This is characterised by selective inhibition of dopaminergic activity in the mesolimbic pathway, with very little inhibition of the nigrostriatal dopaminergic neurons [16,17]. This affinity profile provides antipsychotic effectiveness without the level of extrapyramidal symptoms seen with conventional agents [18].

The present study methodology does not enable us to estimate how many patients within the overall population suffering from schizophrenia would respond in a similarly effective way to sertindole. In addition, the study does not permit us to establish a profile of the patients who may respond particularly well to sertindole, as our sample was small and not representative of the overall population suffering from schizophrenia. It would be interesting for further studies to investigate if these patients who respond particularly well to sertindole have specific clinical characteristics, thus enabling targeted prescription.

## Conclusion

Patients included in this study showed a significant improvement of psychotic symptoms when treated with sertindole after failure of another antipsychotic. This study results have therefore been used for the response to the authorities, as providing evidence that there is indeed a subset of patients who benefit from sertindole after other antipsychotics, including clozapine, have failed.

## Competing interests

JMA has undertaken consultancy work for Lilly, Aventis, Janssen, Lundbeck, Astra Zeneca and BMS; he has received honoraria and hospitality from Lilly, Janssen, Lundbeck, BMS, Pfizer and Novartis in relation to conference presentations on the subject of antipsychotics in schizophrenia.

SM, KH and MT are employees of H. Lundbeck A/S, the manufacturer of sertindole.

## Authors' contributions

JMA participated in the study design and helped to draft the manuscript. SM and KH collected the data, performed the statistical analysis, and drafted the manuscript. MT participated in the study design and statistical analysis. All authors read and approved the final manuscript.

## Pre-publication history

The pre-publication history for this paper can be accessed here:


